# A Pregnant Woman with Spina Bifida: Need for a Multidisciplinary Labor Plan

**DOI:** 10.3389/fmed.2017.00172

**Published:** 2017-10-13

**Authors:** Mary Angela O’Neal

**Affiliations:** ^1^Department of Neurology, Brigham and Women’s Hospital, Boston, MA, United States

**Keywords:** spina bifida, neuraxial anesthesia, pregnancy, complications, labor

## Abstract

Women with spina bifida present both obstetrical and anesthesia challenges. They are more likely to require a caesarian delivery and traditionally neuraxial anesthesia has been avoided due to concerns of worsening neurologic disability. The case of a pregnant woman with a history of a surgically corrected lipomeningocele and tethered cord is presented to illustrate the need for a comprehensive labor plan.

## Introduction

Spina bifida is caused by the failure of the neural tube to close during the first month of embryonic development. There are three main types: spina bifida occulta, meningocele, and myelomeningocele. In spina bifida occulta, the outer part of the vertebrae is not completely closed. The splits in the vertebrae are so small that the spinal cord does not protrude. A meningocele involves a defect in bony closure, which allows the meninges to herniate between the vertebrae. This is a closed spinal dysraphism where the skin is intact, but the underlying spinal cord and associated structures are abnormal. In the patient presented, there was an associated lipomatous mass. Last, in individuals who have a myelomeningocele, the unfused portion of the spinal column allows the spinal cord elements to protrude through the opening. This is an open spinal dysraphism where the malformed spinal cord segment and meninges are not covered by skin.

A tethered cord is defined as an abnormal attachment of the spinal cord to its surrounding tissues. This can occur because of abnormalities in the development in the end of the spinal cord, such as a thickened filum terminale or because of surgery for the spinal dysraphism. The radiologic diagnosis requires a low-lying conus medullaris and a thickened (more than 2 mm) filum terminale. This is distinct from the tethered cord syndrome, which refers to the neurologic symptoms such patients may display. The symptoms typically may include pain, which may be worse with exercise or flexion, lower extremity weakness, and sensory loss and bladder dysfunction (1). Patients with spina bifida often have other associated anomalies of the neural tube particularly Chiari II malformations. This malformation is characterized by cerebellar hypoplasia and caudal displacement of the lower brainstem into the upper cervical canal through the foramen magnum. This leads to impairment of cerebrospinal fluid flow causing hydrocephalus. Chiari II malformations occur in more than 90% of infants with myelomeningocele. Other frequent anomalies include cortical dysplasias and abnormalities involving the corpus callosum and thalami (2).

## Case

A 34-year-old lady G1 P0, 35 weeks pregnant who had a history of surgery for spina bifida and a tethered cord was sent for neurologic evaluation to define both obstetric risks and safety of neuraxial anesthesia. She had a lipomeningocele operated on at 3 months of age. At age 10, she developed a tethered cord syndrome, which required surgery. Unfortunately, due to progressive neurologic symptoms, she required a second untethering procedure. Her neurologic impairments included weakness in legs, distal more than proximal, distal lower extremity numbness, and urinary incontinence. She required braces and crutches to walk and needed to self-catheterize.

### Pertinent Findings on Neurologic Examination

Her right leg was shorter than the left with a smaller right foot. She had bilateral pes cavus deformities and hammer toes. Lower extremity strength: 4/5 hip flexors on the left, 4-/5 on the right, full hip adductors, abductors were 4/5 bilaterally. She had full knee flexion and extension strength. She has no movement of either foot with distal atrophy. Reflexes: 1+ right knee jerk, no left knee jerk, absent ankle jerks. Toes were mute. Sensation showed diminished pinprick right greater than left in an L5-S1 distribution. She could transfer from her wheelchair.

**Figure 1:**
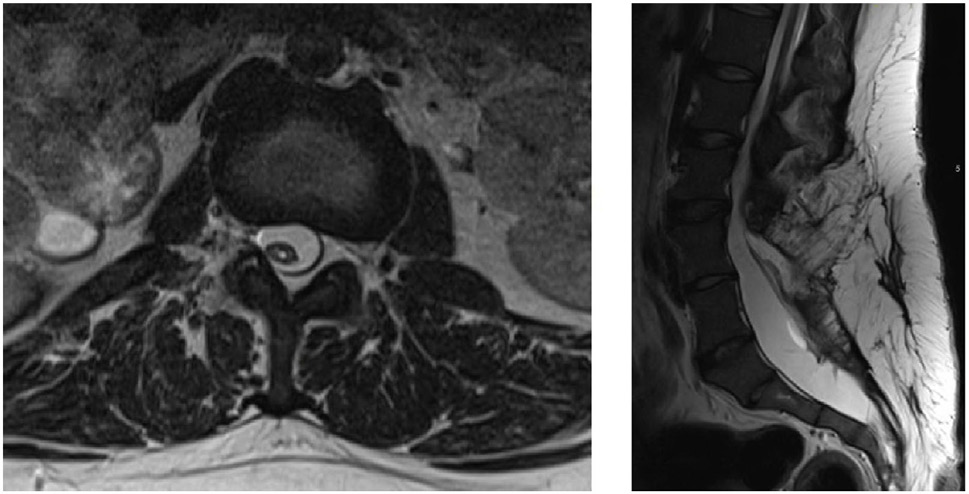
Axial and sagittal T2 lumbar MRI showing postsurgical changes from L3-L5 with neural elements present to the sacrum with an associated syrinx.

Lumbar MRI showed cord dysraphism from L4-L5 with a lipomeningocele. There were surgical changes at L3-L5. Neural elements were present to the sacrum. See Figure 1; obstetrical anesthesia was also consulted. After review of her imaging and joint consultation, the patient was offered epidural anesthesia with the plan to perform the procedure above the level of her previous surgeries. It was explained that due to her prior surgeries that the block might not be as effective and technically difficult. In addition, she understood that it was likely given her significant lower extremity weakness that she would require a caesarian delivery.

She presented at 39 weeks in labor. Epidural anesthesia was successfully placed at T12-L1 using ultrasound guidance. Due to failure to progress, the patient had a caesarian delivery. The epidural anesthesia provided good analgesia. A healthy baby girl was delivered.

## Discussion

### Obstetric Concerns

Fertility is felt to be normal in these women. However, there are no studies looking at fertility in this group of women specifically, so the best information is that extrapolated from studies of women with spinal cord injury (3). In one series of 17 women (14 had a myelomeningocele, 3 had a meningocele), antenatal complications occurred in 14/17 patients. Antenatal admissions were less frequent in women who could ambulate independently as compared with those who were wheelchair dependent. The most frequent complications related to spinal bifida include urinary tract infections, stoma complications, decubitus ulcers, and worsening back pain. Severe thoracic scoliosis in the setting of pregnancy can contribute to restrictive lung disease. Many of these women may have undergone urologic procedures to improve their bladder function. This may complicate performing a caesarian delivery due to the abnormal and complex anatomy. These women also had obstetric complications with a higher frequency of hypertension and preeclampsia. Nearly half of these women required caesarian delivery; this was especially common in women who were wheelchair dependent (4).

### Neuraxial Anesthesia

There are several case reports in the literature of using epidural or combined spinal and epidural anesthesia in women who had prior surgery for their spina bifida. The complications encountered are related to the altered anatomy. These include difficulty locating the epidural space, an asymmetric block likely related to scarring, dural punctures, and the need for multiple operator attempts to place the anesthesia (5). However, reports of permanent neurologic injury after neuraxial anesthesia in such patients are rare (6).

Following surgery in patients, like the one presented, the epidural space is not going to be normal. In addition, even after a detethering procedure, the conus medullaris will likely still be in an abnormally low position (as in our patient). Scoliosis with or without associated corrective surgery can further complicate the spine anatomy. These factors make the ability to perform a safe and efficacious block difficult. Careful analysis of such a patient requires a careful pre-labor analgesic plan. The plan should include a detailed neurologic exam to establish baseline motor and sensory deficits as well as an obstetrical anesthesia consult. The consultants should then discuss the best patient care algorithm.

Imaging is important to look at the anatomy in detail, to locate the best level for neuraxial anesthesia. The most appropriate imaging modality is lumbar magnetic resonance imaging. The level where the cord is not too posteriorly located and the epidural space is likely to be normal is the area where a block should be attempted. Finally, a backup analgesic plan should be formulated in case the block is incomplete or unsuccessful.

## Conclusion

1. Women with spina bifida have an increased risk of obstetric complications. The inability to ambulate is highly correlated with the need for a caesarian delivery.
2. Neuraxial anesthesia can be performed safely and effectively in women with spina bifida.
3. Consultations with neurology and obstetric anesthesia should occur to document a baseline neurologic exam and coordinate an appropriate pre-labor analgesic plan.

## Ethics Statement

Written informed consent was obtained from the patient for the publication of this case report.

## Author Contributions

The author confirms being the sole contributor of this work and approved it for publication.

## Conflict of Interest Statement

The author declares that the research was conducted in the absence of any commercial or financial relationships that could be construed as a potential conflict of interest.
